# Sustained delivery of siRNA/PEI complex from in situ forming hydrogels potently inhibits the proliferation of gastric cancer

**DOI:** 10.1186/s13046-016-0334-y

**Published:** 2016-03-31

**Authors:** Hao Peng, Huawei Yang, Liwei Song, Zheng Zhou, Jinwen Sun, Yunfeng Du, Keyu Lu, Tao Li, Aiguo Yin, Jianhua Xu, Shidong Wei

**Affiliations:** Beijing Meitan General Hospital, 29 South of Xi ba river, Chaoyang District Beijing, 100028 China; The Affiliated Tumor Hospital of Guangxi Medical University, Nanning, 530021 Guangxi Zhuang Autonomous Region China

**Keywords:** siRNA, Collagen hydrogel, Id1, Gastric cancer

## Abstract

**Background:**

Gastric cancer remains a major cause of mortality and morbidity worldwide. In recent years, gene-based therapeutic strategies were confirmed promising in cancer inhibition and attracted great attention. RNA interference (RNAi) is a powerful tool for gene therapy and has been widely employed to aid in treatment for various diseases, especially cancers. However, effective delivery of small interfering RNA (siRNA) to target cells in vivo remains a challenge for that it is prone to degradation and only lasts a few days in rapidly dividing cells.

**Methods:**

Due to its biocompatibility and well-established safety profile, collagen represents a favourable matrix for in-site drug delivery. In the study, collagen hydrogel was used as carriers to test the feasibility of localized and sustained delivery of Id1-targeted siRNA for in vivo gastric cancer inhibition. To enhance the siRNA delivery, cationic polyethylenimine (PEI) was further emplored for scallold modification. The efficacy of siRNA delivery and cancer inhibition were evaluated with multimodality of mehods in vitro and in vivo.

**Results:**

Our results showed that addition of polyethylenimine (PEI) to collagen can facilitate entry of Id1-siRNA into target cells, prolong the silencing effect, and further inhibit tumor growth both in vitro and in vivo.

**Conclusion:**

This collagen-based delivery system may facilitate the pathogenesis elucidation and design of effective therapies against gastric cancer.

## Background

RNA interference (RNAi) is a post-transcriptional gene silencing tool that can inhibit the expression of target gene by causing degradation of the specific mRNA molecule. As a new technology, it is widely used in functional research on cancer genes as well as therapeutics for a variety of tumors [1]. In gastric cancer, several genes have been proved to be closed related to proliferation and migration of gastric cancer cells, such as Stathmin1, Id1, PLCE1. These genes have been shown to be important targets for gastric cancer therapy for the fact that silencing of them by RNAi significantly inhibits proliferation and migration of gastric cancer cells [2–4]. However, effective delivery of small interfering RNA (siRNA) to target cells in vivo is far from realizing its full therapeutic potential because it is prone to degradation by RNases [5], the silencing effect only lasts a few days in rapidly dividing cells [6], and retention of the siRNA at a specific location is difficult [7].

Lentiviral transfection was effective to deliver siRNA into target cells, but safty will be a major concern when considerred in vivo application. No-viral biomaterial vectors should be more rational option for in vivo siRNA delivery. In fact, several biomaterials have been investigated as scaffold for in vivo delivery of therapectic agents, such as carbon nanotubes and chitosan [8, 9]. As a major natural constituent tissue and a major structural protein of any organ, collagen is of particular interest as a biomaterial in drug delivery system. Biomaterials made of collagen possess the advantages of biocompatibility, non-toxicity, and well-documented properties [10]. Collagen has been shown to retain siRNA locally and release it in a sustained manner to prolong the effect directly at the specific site by Krebs and his colleagues [11]. Through this delivery system, novel candidates can be tested for effective therapies against gastric cancer before further clinical application. In addition, characteristics of this strategy for interference and therapeutics on gastric cancer, both in vitro and in vivo, needs to be demonstrated.

In this study, we mainly investigated the feasibility of localized and sustained delivery of Id1-targeted siRNA incorporated within collagen, the characteristic of siRNA release profile, and its effect on growth and migration ability of gastric cancer cells both in vitro and in vivo.

## Methods

All the experiments in the study were approved by the ethics committee of Beijing Meitan General Hospital (China).

### Cell culture

The SGC-7901 gastric cancer cell line was purchased from ATCC and cultured in high-glucose DMEM (Gibco, BRL, Beijing, China) supplemented with 10 % fetal bovine serum at 37 °Cwith 5 % CO_2._

### Id1 small interfering RNA (siRNA)

Id1-specific siRNA used for Id1 knockdown and the control siRNA were synthesized by Invitrogen (Beijing Invitrogen Co., Ltd.). The sequences of siRNA targeting the Id1 coding region were as follows: sense, 5’-CUCGGAAUCCGAAGUUGGADTDT-3’ and antisense, 5’-UCCAACUUCGGA UUCCGAGDTDT-3’. The siRNAs were transfected into cells by Lipofectine 2000 (Invitrogen, USA), according to the manufacturer’s instructions.

### MTT assay

Briefly, cells were trypsinized and seeded into 96-well plates at a density of 5 × 10^3^ cells/well in a volume of 150 μl. The cells were incubated with 20 μl, 5 mg/ml of MTT (Sigma-Aldrich, St. Lousis, MO, USA) solution for 4 h under regular culture condition. After the supernatant was removed, 150 μl DMSO was added to dissolve the crystals. The absorbance values at 570 nm were read at specified time points with a BioTek Synergy 2 multiwell spectrophotometer. Viable cells were tested at 0, 1, 2, 3 days after plating, and each experiment was repeated three times.

PureCol collagen (97 % type I collagen) was obtained from Inamed Biomaterials (Fremont, CA). Polyethylenimine (PEI) “Max” was obtained from Polysciences, Inc. (Warrington, PA). The RiboGreen RNA quantitation reagent was obtained from Invitrogen (Carlsbad, CA).

### Release and bioactivity of siRNA from hydrogels

Hydrogels containing 15 μg (10 μl) siRNA were fabricated in transwell membranes with 0.4 μm pore-size. The siRNA was mixed into 90 μl of 3 mg/ml collagen solutions. The collagen solution was kept on ice during this process. For collagen mixed with PEI, PEI was mixed with siRNA to form a final concentration of 0.2 mg/ml at room temperature for 20 mins . Then the mixture was added into the same amount of collagen solution and pipetted onto transwell membranes. The collagen solution was placed into a 37 °C incubator for 45 min to allow hydrogel formation. For release studies, the transwell membranes were placed into the wells of a 24-well plate containing PBS, the PBS was replaced at various time points, and the siRNA content in each sample was measured using the RiboGreen RNA quantitation reagent.

### Transfection of cells incorporated within hydrogels

The SGC-7901 gastric cancer cells were mixed within the collagen solution at a density of 5 × 10^5^ cells/ml. The hydrogels on the transwell membranes were cultured in the presence of accell delivery media (ADM), which was replaced with ADM supplemented with 1 % FBS on days 1, 3, and 5. The cells within the hydrogels were trypsinized by type I collagenase and the DNA content was measured by Quant-iT™ PicoGreen Kit (Invitrogen).

### RT-PCR and western blotting

Total RNA from cultured cells was extracted and then reverse transcribed using commercial kits from Tiangen (Beijing, China). Then, 2 μl of cDNA was used for the quantitative polymerase chain reaction (qPCR) using SYBR Green Realtime PCR Master Mix (TOYOBO, Osaka, Japan) in Eppendorf Mastercycler Realplex Real-time PCR system. GAPDH was used for normalization of mRNA. Cells ere collected and treated with RIPA lysis buffer. Lysate with 60 μg protein was separated by 15 % sodium dodecyl sulphate-polyacrylamide gel electrophoresis. After electrophoresis, proteins were transferred onto a polyvinylidene difluoride (PVDF) membrane (Millipore, Billerica, MA, USA) and blocked with 5 % milk. Then the membrane was incubated with anti-cyclin D1, p16, p-Akt, Akt and GAPDH antibodies (Cell Signaling Technology) overnight at 4 °C, followed by washing and incubation with a horseradish peroxidase-conjugated secondary antibody (Santa Cruz Biotechnology). The membrane was washed and detected by enhanced chemiluminescence with Millipore reagents. The expression level of the target protein was normalized to GAPDH by a scan software.

### Studies of gastric cancer xenograft tumor models in nude nice

Six-week-old male BALB/c nude mice were purchased from laboratory animal center of Academy of Military Medical Sciences and housed in a temperature-controlled, pathogen-free animal facility with 12-h light and dark cycles. 2 × 10^6^ gastric cancer cells were mixed with DMEM solution, siRNA solution, siRNA solution within collagen, siRNA solution within collagen and PEI, respectively and then injected hypodermically into the nude mice. After 4 weeks, the tumors were collected and weighted.

### Immunocytochemistry

Expression of cyclin D1, P21 and PCNA in tumor cells were identified by immunostaining with a polyclonal rabbit anti-rodent antibodies. Diamino-benzidine and alkaline phosphatase substance (ZhongShan Goldenbridge biotech, Beijing, China) were used to visualize the proteins.

### Statistical analysis

Data were expressed as the means ± SD. Statistical analyses were performed using Student’s *t*-test. *P* < 0.05 indicated statistical significance.

## Results

### Effects of Id1 siRNA or pcDNA3.1-Id1 on cell cycle-related protein expression and SGC-7901 cell proliferation

**Fig. 1:**
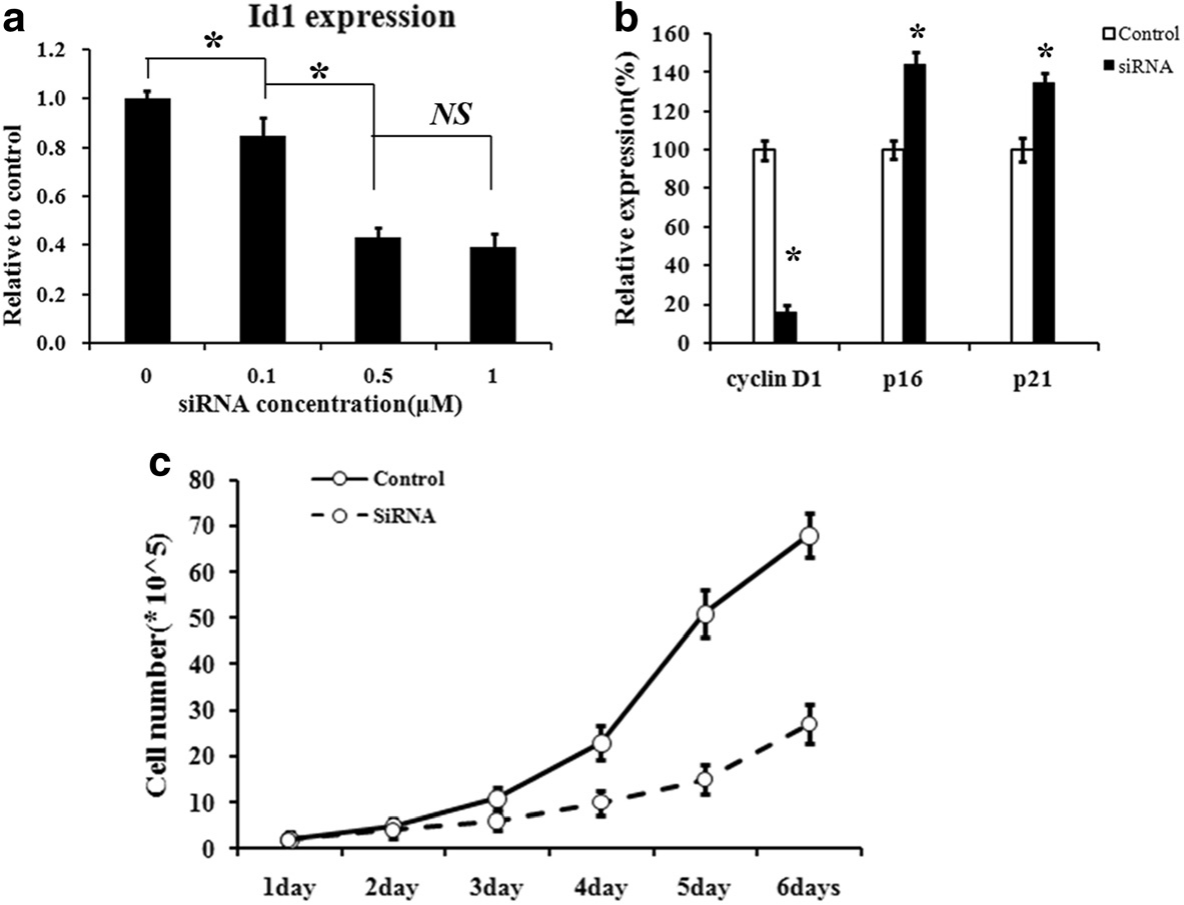
Concentration optimization of siRNA for efficient interference on target gene. **a**. RT-PCR analysis showed that Id1 expression can be suppressed by siRNA in a dose-dependent manner. 1 μM concentration of siRNA was used in the followed assays for sufficient interference effect. **b**. Effects on the cell cycle-related genes through downregulation of Id1. **c**. MTT assay was performed indicating that Id1-siRNA showed remarkable inhibitory effects on the proliferation of SGC-7901 cells

The effects of siRNA or pcDNA3.1-Id1 on the levels of Id1, cyclin D1, p16, p21 were evaluated using reverse transcriptase-PCR. As shown in Fig. 1a, the target gene expression decreased with the dose of Id1 siRNA. When the concentration of Id1 siRNA reached 0.5 μm or more, the maximal inhibition of target gene Id1 in SGC-7901 cells was achieved. Therefore, 1 μm concentration of Id1 siRNA was chosen for the following experiments. Then, we detected the expression of Cell Cycle genes. As shown in Fig. 1b, cyclin D1 mRNA was found to be decreased due to the silencing of Id1, while p16 and p21 expression were increased in the siRNA tranfected cells. The results suggested that cell proliferation may be inhibited. To evaluate the effect of Id1 on the proliferation of SGC-7901 cells, MTT assay was performed. As shown in Fig. 1c, proliferation of SGC-7901 cells was inhibited by Id1 interference and the effect was evident after 72 h transfection with Id1 siRNA.

### Cumulative release of siRNA from hydrogels

**Fig. 2:**
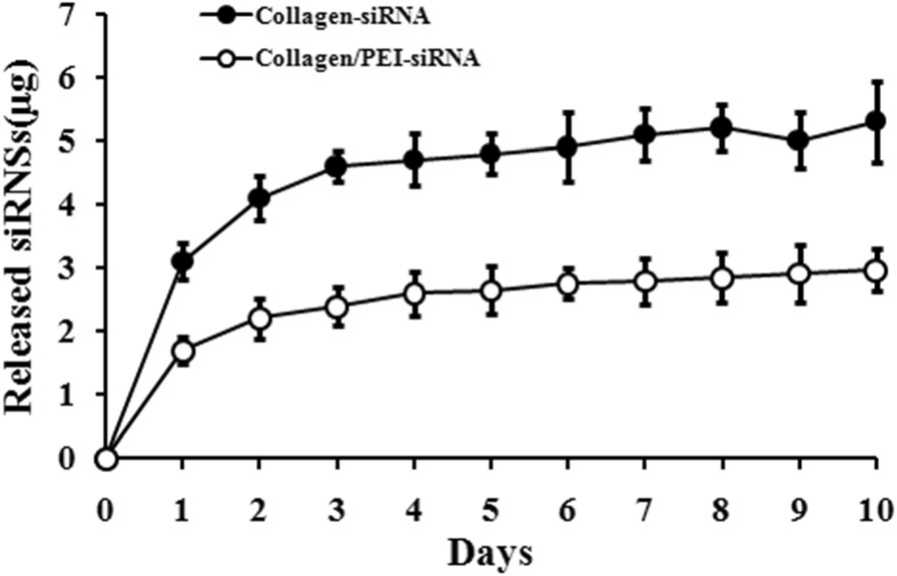
Cumulative release of siRNA from hydrogels. Collagen with or without the addition of PEI to modulate siRNA release

To measure the release of siRNA molecules from collagen hydrogels, the same amount of siRNA was incorporated into collagen or collagen with PEI, release from two systems was quantified over a period of 10 days. As shown in Fig. 2, siRNA was released in a sustained manner for about 2 weeks from collagen. And the addition of PEI to collagen delayed the release of siRNA into the surrounding media. These results suggested that siRNAs were effectively retained within the hydrogels, which may decrease their loss and enhance their efficacy to surrounding cells.

### Target gene inhibition by siRNA

**Fig. 3:**
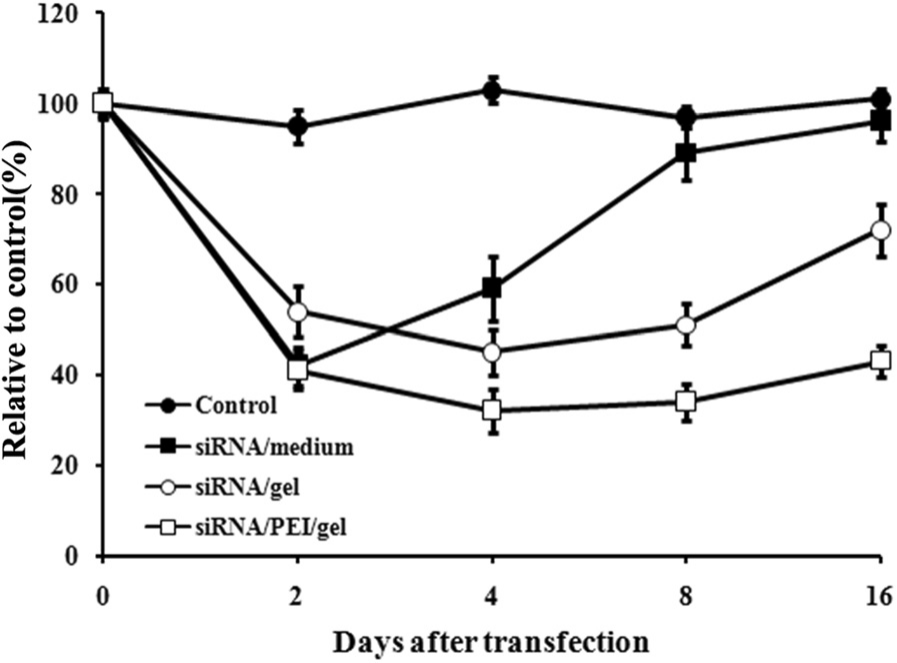
Different release profiles of siRNA under different conditions. Cells were exposed to no siRNA, siRNA only in the hydrogels, siRNA present in the media, or siRNA in hydrogels with the addition of PEI. RT-PCR analysis was performed. Values are the means ± SD of 3 wells. Id1 mRNA levels in the treated-cells were normalized to the control group cells

lipofectamine 2000 was used as control for comparison. As shown in Fig. 3, the maximal inhibition of target gene through lipofectamine 2000 was achieved at 2 days after tranfection and then, the inhibited Id1 expression gradually recoverred. The inhibitory effect through lipofectamine 2000 tranfection only lasted less than one week; while those for siRNA incoporated into collagen lasted for 2 weeks. The maximal inhibition of target gene was achieved at 4–8days, and the inhibiton was more potent than tha by lipofectamine 2000 tranfection. The addition of PEI into collagen further prolonged the inhibitory effect, which may indicate that PEI delayed the release of siRNA into the surrounding media. Meanwhile, the could exclude the degradation or inactivation of siRNA within the complex.

### Measurement of tumor cell DNA content and cell proliferation analysis

**Fig. 4:**
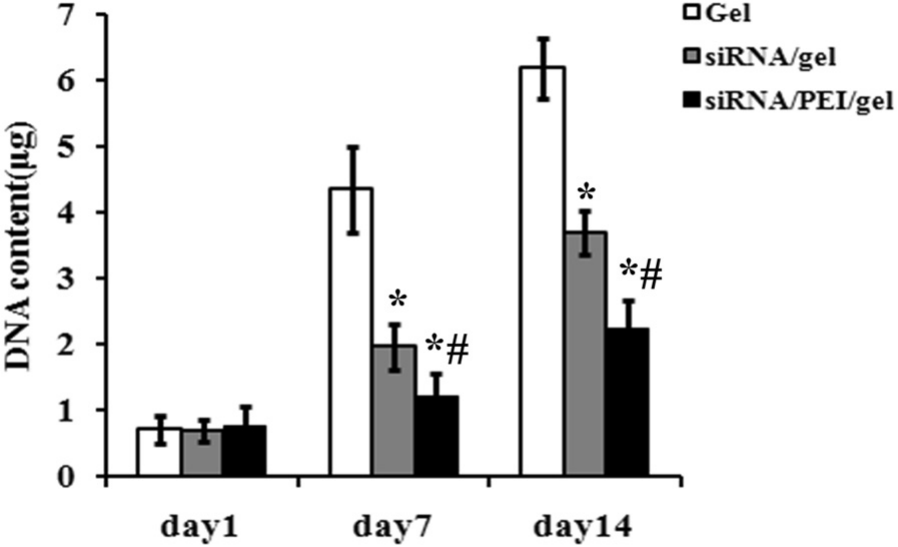
Cell proliferation analysis through measuring DNA content from tumor cells. Cells were exposed to no siRNA, siRNA only in the hydrogels, or siRNA in hydrogels with the addition of PEI and DNA content was measured

To determine cell proliferation, DNA content was measured to quantify the cell numbers. As shown in Fig. 4, the DNA content from cells exposed to siRNA/collagen and siRNA/collagen/PEI was significantly lower that of the control group both at day 7 and day 14, indicating that tumor cell proliferation was inhibited.

### Western blotting and expression level changes of cell cycle-related genes from different groups

**Fig. 5:**
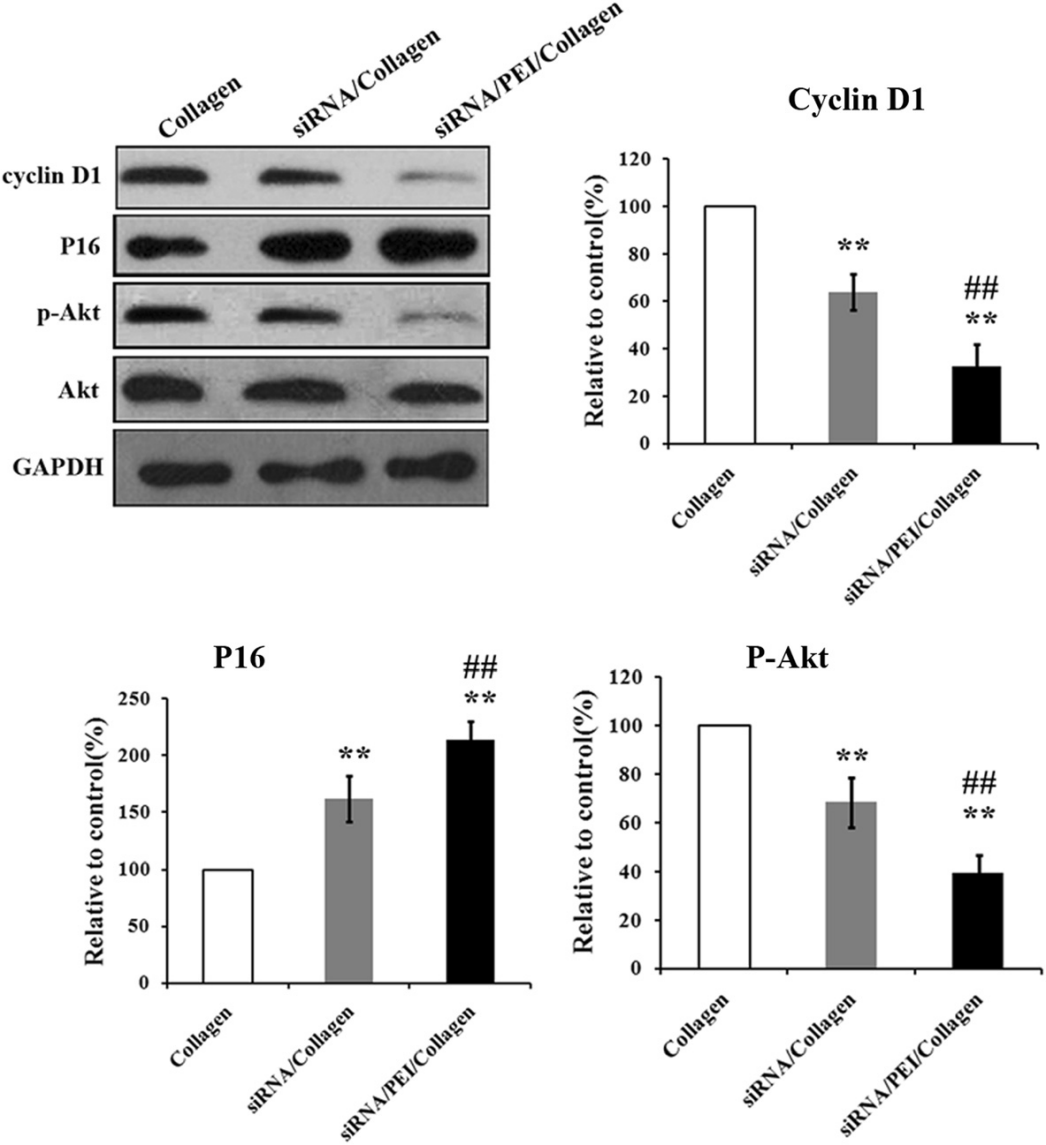
The expression level changes of cell cycle-related genes from different groups. Western blot analysis of the expression levels of cyclin D1, p16 and p-Akt. The expression of the target proteins were expressed as relative levels to GAPDH

The changes of certain cell cycle regulators were analyzed through western blotting. As shown in Fig. 5, The expression levels of cyclin D1 and p-Akt were decreased significantly in cells exposed to siRNA in the hydrogels compared comtrol, while p16 was increased, which was consistent with previous studies [12]. Further, the addition of PEI strengthens this effect.

### Inhibitory effect on tumor growth in vivo

**Fig. 6:**
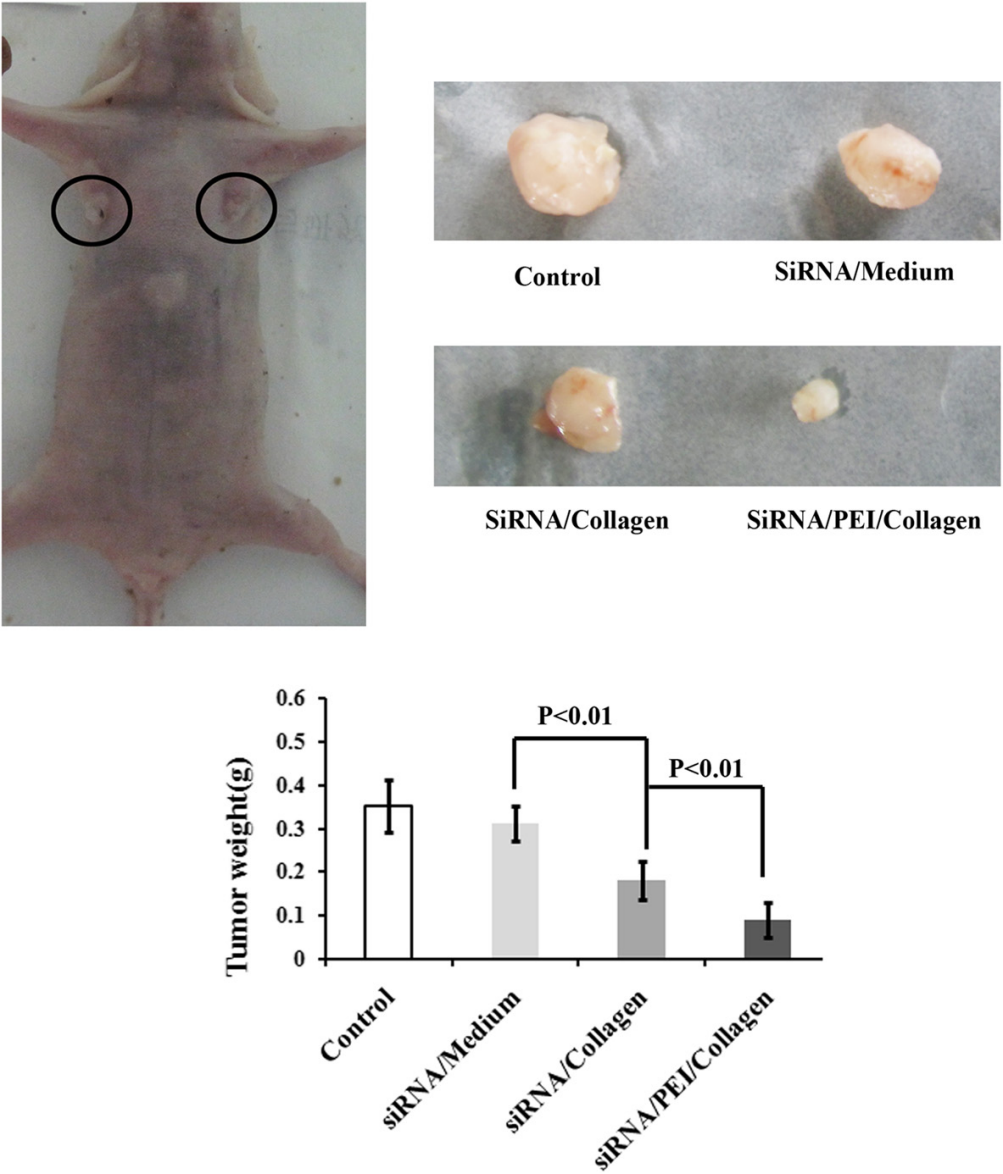
Inhibitory effect on tumor growth in vivo. Macroscopic images of the tumors from nude mice and tumor weight from different groups

Four weeks after transplantation, tumors were collected and their weights were measured. As shown in Fig. 6, the tumor volums were obviously smaller in siRNA/collagen and siRNA/collagen/PEI groups compared with control group. Weighing of tumors also demonstrated that tumor weight from siRNA/collagen and siRNA/collagen/PEI treated nude mice were significantly lower than that from the control group, which showed that collagen-mediated delivery of siRNA inhibits tumor growth in vivo.

### Immunostaining of cyclin D1 and P21 in tumor cells

**Fig. 7:**
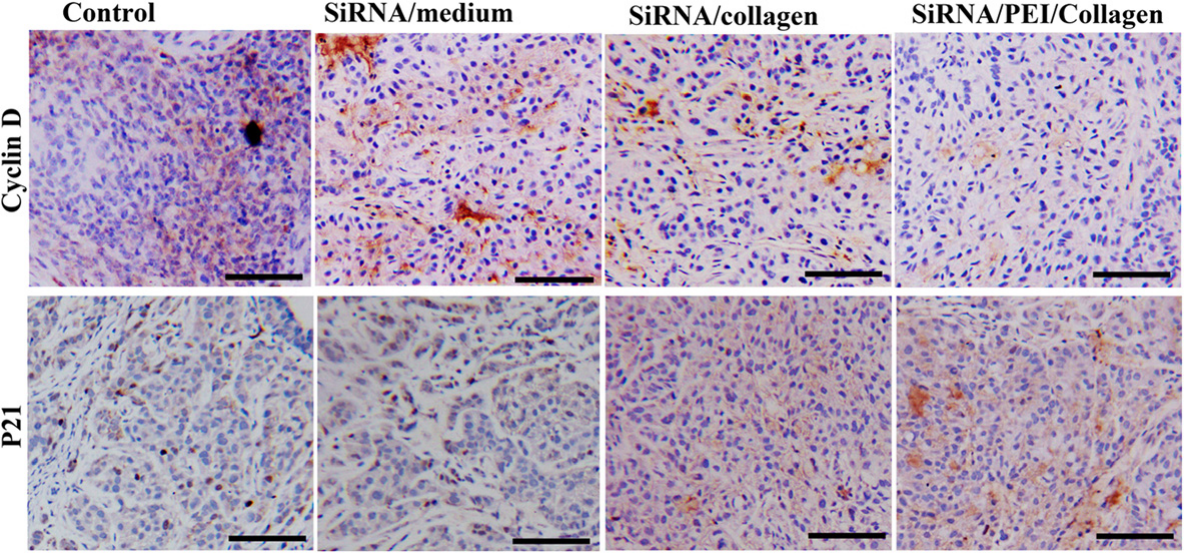
Immunostaining of cyclin D1 and P21 in tumor cells. cyclin D1 expression was downregulated in the siRNA/collagen treated tumor cells, while P21 expression was significantly increased, indicating that the cell cycle was inhibited. The inhibitory effect from the siRNA/PEI/collagen group was more evident. (scale bars, 100 μm)

To determine the effects of siRNA transfection on the cell cycles within tumors, paraffin-embeded sections were prapared and immunostained with cell cycle-related antibodies. As shown in Fig. 7, significantly less cells were stainned positive by anti-cyclin D1 antibodies in the siRNA/collagen treated tumor cells, suggesting that cyclin D1 was significantly downregulated; while P21 expression was significantly increased, indicating that the cell cycle was inhibited. The inhibitory effect from the siRNA/PEI/collagen group was more evident.

### Immunohistochemical analysis of PCNA expression in tumors from different groups

**Fig. 8:**
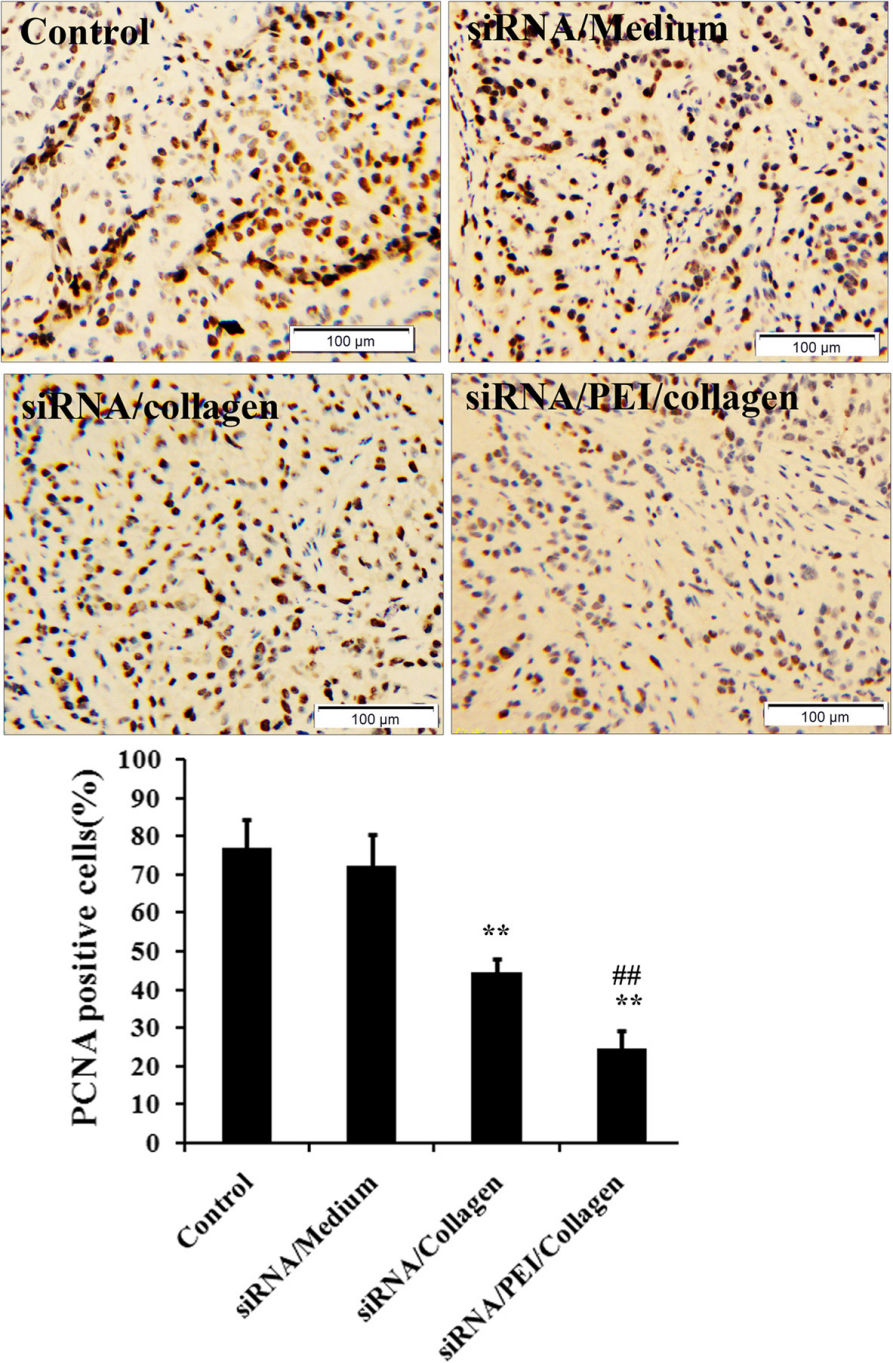
Immunohistochemical analysis of PCNA expression intumors from different groups. The expression rate from siRNA/collagen group and siRNA/PEI/collagen group was significantly lower than that from control group and siRNA/Medium group. And the inhibitory effect on cell proliferation from the siRNA/PEI/collagen group was the most evident. **P < 0.01 compared with control group; ## *P* < 0.01 compared siRNA/collagen group

PCNA was an important marker of cell proliferation. In addition, its expression has also been suggested to have prognostic significance for gastric cancer [13]. To determine the cell proliferation within tumors, we further performed immunostaining on tumor sections with PCNA antibodies. As shown in Fig. 8, the positive immunostaining rate of PCNA for siRNA/collagen group and siRNA/PEI/collagen group was significantly lower than that of the control group and siRNA/Medium group, indicating cell proliferation was significant inhibited. In comparison, the inhibitory effect on cell proliferation from the siRNA/PEI/collagen group was the most evident.

## Discussion

RNA interference (RNAi) is a biological process in which introduction of double-stranded RNA (dsRNA) into cells can effectively and specifically lead to the degradation of corresponding mRNAs [14]. This powerful tool has been widely employed to identify gene functions, elucidate signal pathways, and aid in treatment for various diseases, especially in cancers. However, effective delivery of small interfering RNA (siRNA) to target cells in vivo remains a challenge for that it is prone to degradation and the silencing effect only lasts a few days in rapidly dividing cells. Thus, methods for localized and sustained delivery of silencing RNA into cells need exploring. In the present study, we first employed collagen hydrogel as carriers for in vivo delivery of siRNA into gastric tumors. We demonstrated that siRNA could be effectively retained within the hydrogel due to its *in situ* gelation property. More importantly, the incorporated siRNAs could be of delayed release, which significantly prolonged their action time and enhanced their efficacy on target gene. Consequently, a potent inhibition of gastric tumors was achieved in vivo through collagen-based siRNA delivery. The findings and methods of the study may indicate a promising strategy for gene therapy of cancers in future.

Current approaches for delivery of siRNA into target cells include viral-based transfection, incorporation into liposomes, chemical conjugation with molecules to facilitate targeting, complexation with positively charged peptides or polymeric nano- or microspheres [15]. Generally, these methods could be divided into two categories: viral method and non-viral method. A typical viral method for siRNA delivery should be lentiviral transfection, which was a lentivirus-based method to deliver siRNA into target cell. In past years, lentiviral transfection of siRNA was widely used and for many types of cells, it was effective for siRNA delivery. However, lentiviral transfection was companied with potential safety concerns, such as insertional mutagenesis and aberrant splicing [16, 17]. These were important reasons why non-viral methods were extensively investigated. In the field of non-viral siRNA delivery, most methods were based on compatible biomaterial vectors. Collagen is a native extracellular matrix molecule which can serve as physical support to promote tissue organization and scar tissue formation [18, 19]. It has been demonstrated by different groups that collagen hydrogel were favourable vectors, that they have the potential to deliver various bio-agents, such as cytokines [20], living cells [21, 22], as well as exogenous microRNAs [23]. One strong advantage of this hydrogel as biopolymer scaffolds is that it is injectable and delivery to the site of interest is minimally invasive. Also, its hydrophilic nature and high gas permeability permits easy transport of nutrients and oxygen and removal of waste products. Thus this injectable biopolymer-based siRNA delivery system may have great utility in therapeutic medicine.

Different from certain Western European countries and the United States, gastric carcinoma is a common disease with high incidence rates in several Asian countries, particularly in Japan and China [24, 25], and the 5-year survival rate is low due to the majority of the cases being detected at advanced stages [26]. Finding new targets to improve therapeutic or preventive strategies is important. Inhibitor of DNA binding 1 (Id1) is a member of the helix-loop-helix transcription factor family that is overexpressed in various types of cancer, including gastric carcinoma [27]. Previous studies showed that Id1 is a prognostic marker in patients with gastric cancer which is involved in the growth and migration of gastric cancer cells [28, 29]. Certain reports have suggested that Id1 can regulate various cell processes, including proliferation, apoptosis, cell cycle, differentiation and angiogenesis [3, 30, 31]. Further research have showed that down-regulation of Id1 by small interfering RNA in gastric cancer inhibits cell growth via the Akt pathway [32]. Thus, Id1 may be an important target for gastric cancer therapy. In the study, we chose Id1 as target gene aiming to investigate the inhibitory efficacy of collagen-based in vivo siRNA delivery on gastric cancer. We confirmed that this delivery system was effective for in vivo gene silencing. In addition to Id1 gene, several other genes have also been confirmed to be related to the proliferation, migration and survival of gastric cancer cells, such as Class I phosphoinositide 3-kinase, stathmin1, PLCɛ1 [2, 33, 34], and so on. Though other genes were not tried in the study, we believed that the delivery system should be equally effective for other genes. Actually, in an in vitro study by Krebs and colleagues [11], the delivery system was confirmed effective too with another target gene.

## Conclusions

In this study, we mainly investigated the feasibility of localized and sustained delivery of Id1-targeted siRNA which has been incorporated into collagen and its effect on the growth and migration ability of gastric cancer cells both in vitro. Results showed that the release profile of siRNA incoporated into collagen was significantly prolonged. And the addition of polyethylenimine (PEI) to collagen can facilitate the entry of siRNA into target cells which further prolong the silencing effect of siRNA. Also this collagen-based delivery system exhibits evident inhibitory effect on tumor growth in vivo, which may be further utilized for interference and therapeutics on gastric cancer cells.
